# Quantitative PCR Assays for the Strain-Specific Identification and Enumeration of Probiotic Strain *Lacticaseibacillus rhamnosus* X253

**DOI:** 10.3390/foods11152282

**Published:** 2022-07-30

**Authors:** Lei Zhao, Dong Zhang, Yang Liu, Yi-Nan Zhang, Dong-Qing Meng, Qiong Xu, Jiang Zhong, Qiu-Yue Jiang, Yu Zhao, Shi-Jie Wang

**Affiliations:** 1 Key Laboratory of Milk and Dairy Products Detection and Monitoring Technology for State Market Regulation, Shanghai Institute of Quality Inspection and Technical Research, Shanghai 200233, China; zhaolei19890117@163.com (L.Z.); zhangyn@sqi.org.cn (Y.-N.Z.); xuqiong@sqi.org.cn (Q.X.); 2 Department of Microbiology and Microbial Engineering, School of Life Sciences, Fudan University, Shanghai 200438, China; jzhong@fudan.edu.cn (J.Z.); 15110700011@fudan.edu.cn (Q.-Y.J.); 3 Junlebao Dairy Group, Shijiazhuang 050221, China; zhangdong18540@jlbry.com; 4 College of Life Sciences, Shanghai Normal University, Shanghai 200234, China; mdq18238002083@163.com (D.-Q.M.); zhaoyu@shnu.edu.cn (Y.Z.)

**Keywords:** probiotics, quantitative PCR, single nucleotide polymorphisms, strain-specific identification, enumeration

## Abstract

Probiotics are universally recognized for their health benefits, despite the fact that their effects depend on the strain. Identification and enumeration of probiotic strains are required prior to evaluating their effectiveness. *Lacticaseibacillus rhamnosus* X253 is a potential probiotic strain with antioxidant capacity. Comparative genomics and single nucleotide polymorphisms (SNPs) were used to identify a strain-specific locus within the *holA* gene for strain X253 that was distinct in 30 different *L. rhamnosus* strains. Using quantitative PCR, the primers and probe designed for the locus were able to distinguish *L. rhamnosus* X253 from the other 20 probiotic strains. The chosen locus remained stable over 19 generations. The sensitivity of the assay was 0.2 pg genomic DNA of *L. rhamnosus* X253, or 10^3^ cfu/mL bacteria of this strain. In terms of repeatability and reproducibility, relative standard deviations (RSD) were less than 1% and 3%, respectively. Additionally, this assay achieved accurate enumerations of *L. rhamnosus* X253 in spiked milk and complex powder samples. The strain-specific assay could be used for quality control and compliance assessment of dairy products.

## 1. Introduction

Probiotics are microbes that, when taken in appropriate quantities, have beneficial effects on the host and contribute significantly to maintaining balance in the gut microbiome [1]. In recent years, some studies on the “gut–brain axis” and “gut–liver axis” have shown that probiotics can prevent and cure diseases by promoting two-way communication between the intestine and other organs [2,3]. Probiotics such as *Lacticaseibacillus rhamnosus*, *Lactiplantibacillus plantarum*, *Lactobacillus acidophilus*, and *L. casei* are routinely employed in dairy products [4].

*L. rhamnosus* has been utilized as a kind of probiotic bacteria for over 30 years, and its efficacy has been demonstrated in several studies [5]. It has been shown to be able to tolerate and colonize the human gastrointestinal tract [6], inhibit *Salmonella typhimurium* growth [7], alleviate antibiotic-associated diarrhea [8], remove cadmium and aflatoxin B1 from aqueous solution [9], enhance the immune response [10], and attenuate allergic reactions [11].

Notably, the probiotic activities are strain-specific in terms of safety and functioning. According to guidelines of both the World Health Organization (WHO) and the European Food Safety Authority (EFSA) regarding the evaluation of probiotics, the probiotic strains and the number of active bacteria in products are required to be listed on the label [1,12]. Accurate strain identification is crucial for the quality and efficacy of probiotic foods. Due to the inability of 16S rDNA or protein-coding genes to distinguish between strains of the same species [13], a variety of DNA fingerprinting approaches, including pulsed field gel electrophoresis (PFGE), amplification length polymorphism (AFLP), and random amplified polymorphic DNA (RAPD), have been used to identify probiotic bacteria at the strain level [14]. However, since these approaches are inefficient, difficult to repeat, and require a high level of skill and equipment, they are inappropriate for rapid strain-specific identification [15]. Genomics and comparative genomics provide a powerful approach for developing novel strain-level identification tools. Single-nucleotide polymorphism (SNP) refers to DNA sequence polymorphism generated by variation in a single nucleotide, which is genetically stable and could serve as the basis for strain-level specificity in probiotics [16]. 

Furthermore, in order to achieve the predicted health benefits, an adequate number of probiotics must be consumed [17]. The immune system can only be activated when the density of probiotic cells in the solution exceeds 10^6^ cfu/mL [18]. Probiotic products are monitored for the number of bacteria cells throughout the manufacturing process, and the results are incorporated into the formulation of multi-strain products [19]. Therefore, accurate counting is necessary to evaluate the therapeutic properties of probiotics as well as to ensure the quality of products. With its high specificity, consistency, and extensive equipment availability, quantitative PCR facilitates the development of reliable and effective assays to detect specific probiotic strains.

*L. rhamnosus* X253 is a safe probiotic strain isolated from fermented milk in Xinjiang, China, with good tolerance to human gastrointestinal fluids and antioxidant capacity. Its genome has been sequenced and uploaded to the NCBI database (accession number CP073711). In this study, a locus of the X253 strain was identified by comparative genomic and SNP analysis. Using quantitative PCR, an efficient and stable method for detecting and quantifying the X253 strain was developed, and its specificity, stability, sensitivity, repeatability, and reproducibility were validated. The method facilitated the rapid determination of a specific strain in complex dairy products.

## 2. Materials and Methods

### 2.1. SNP Analysis

The genomes of 30 *L. rhamnosus* strains were downloaded from the GenBank database (Table 1), and the sequences of other 29 *L. rhamnosus* genomes were aligned to the sequence of *L. rhamnosus* X253 using the Artificial Fastq Generator (version 3.0, https://sourceforge.net/projects/artfastqgen/, accessed on 15 April 2022) [20] and Bwa software (version 0.7.17, http://bio-bwa.sourceforge.net/, accessed on 20 April 2022) [21]. After sorting the data and removing duplicate sequences using Samtools (version 1.6, https://github.com/samtools/samtools) [22] and Samamba (version 0.5.0, https://www.open-bio.org/wiki/Sambamba, accessed on 20 April 2022) [23], a total of 12 specific SNP loci were obtained for the X253 strain (Table S1). The analysis was completed by Amplicongene (Shanghai, China). 

### 2.2. Extraction of Nucleic Acids

The DNA of bacterial cultures and probiotic products was extracted using the Bacterial Genomic DNA Extraction Kit (Tiangen Biotech, Beijing, China) according to the manufacturer’s instructions. The DNA was eluted in 50 µL of elution buffer. The purity of DNA was analyzed with UV absorbance on a Nanodrop 2000 Spectrophotometer (Thermo Fisher Scientific, Wilmington, MA, USA), the DNA with OD_260/280_ ratios of 1.8 to 2.0 was prepared for further quantification. A Qubit 3.0 Fluorometer (Life Technologies, Carlsbad, CA, USA) and a dsDNA HS Assay Kit (Life Technologies, Carlsbad, CA, USA) were used for DNA quantification [24,25]. The samples were kept at −20 °C.

### 2.3. Design of Quantitative PCR Primers and Probes 

The primers and probes for the 12 specific SNP loci of *L. rhamnosus* X253 were designed using the Primer Express 3.0 software (Applied Biosystems, Foster City, CA, USA). After experimental confirmation, the SNP locus on the *holA* gene was selected as the specific locus for further testing because of its high specificity and low Cq values. Primers and probe utilized in the tests were synthesized by Sangon (Shanghai, China) (Table 2). 

### 2.4. Specificity

The specificity of primers and probe was studied with 21 distinct bacteria strains, including 6 strains of *L. rhamnosus*, and 15 strains of other lactic acid bacteria (Table 3). For specificity testing, DNA concentrations in all samples were normalized to 10 ng/µL. The quantitative PCR assays were performed on a LightCycler^®^ 480 platform (Roche, Basel, Switzerland). Each PCR reaction (20 µL total volume) consisted of 10 µL of Premix Ex Taq™ (TaKaRa, Beijing, China), 1 µL of forward primer (5 µM), 1 µL of reverse primer (5 µM), 1 µL of probe (10 µM), 1 µL of DNA template, and 6 µL of sterile water. PCR cycling conditions were denaturation at 95 °C for 2 min, and 40 cycles of amplification (95 °C for 5 s, and 52 °C for 35 s). Sterile water was used in place of DNA templates as a blank control, and each experiment was conducted in triplicate.

### 2.5. Stability during Passage

We extracted DNA from 10^8^ cfu/mL of *L. rhamnosus* X253 subcultured bacterial cells of generations 5, 9, 15, and 19. To determine the variability of Cq values, DNA concentrations in each sample were normalized to 10 ng/µL before quantitative PCR amplification on a LightCycler^®^ 480 platform.

### 2.6. Sensitivity and Efficiency

Sensitivity tests on DNA concentration and cell density were performed. The DNA-based sensitivity was tested by five tenfold serial dilutions from three different starting DNA concentrations (10, 5, and 2 ng/µL) [26], while the cell density-based sensitivity was measured by extracting nucleic acid from cultures of different densities (10^8^–10^2^ cfu/mL). All tests were carried out in triplicate using the quantitative PCR protocol described above on a LightCycler^®^ 480 platform. Standard curves between the Cq values and logarithmic DNA concentration or between the Cq values and logarithmic cell density were generated using GraphPad Prism 6. The slope and R squared values were calculated using GraphPad Prism 6 as well. The ThermoFisher qPCR efficiency calculator was used to compute reaction efficiency from slope values.

### 2.7. Repeatability and Reproducibility

The repeatability of the test was determined by repeating the assay over a short period of time, and the reproducibility of the test was determined by doing the assay on two different quantitative PCR platforms (LightCycler^®^ 480 and ABI7500). Five samples were examined for repeatability and reproducibility at three different DNA concentrations (0.1, 1, and 10 ng/µL). The results of repeatability and reproducibility were represented as relative standard deviations (RSD), which were calculated as the mean Cq of the same sample measured at different times, or on different platforms, respectively.

### 2.8. Spiked Sample Assay

To assess the viability of the quantitative PCR approach in a milk matrix, we established another standard curve using bacterial DNA isolated from artificially spiked milk. A serial 10-fold dilution (10^7^ to 10^2^ cfu/mL) of X253 culture was carried out using commercial fermented milk (including *L. plantarum*, *L. bulgaricus*, *Streptococcus thermophilus*, and *L. acidophilus*) as the substrate. For DNA extraction, 2 mL of each dilution was centrifuged at 1000 rpm for 5 min, then 1 mL of the supernatant was transferred to a new EP tube, centrifuged at 12,000 rpm for 10 min, and the precipitate was collected. The DNA was amplified by quantitative PCR to measure the resistance of the assay to interference from other strains and substrates.

### 2.9. Actual Product Assay

Five batches of multi-strain bacterial powder samples, including oligosaccharides and excipients, were employed in the actual product assay. The powder was made up of *L. rhamnosus* X253, *Bifidobacterium animalis subsp. lactis* Bb12, *L. paracasei* N115, and *L. acidophilus* NCFM. Sample preparation was carried out according to the method of the Chinese National Standard (4789.35-2016). The 25 g product sample (A) was dissolved in 225 mL (B) of sterile normal saline and diluted 10-fold with sterile normal saline and the dilution times (C) were recorded. The X253 culture at 10^9^ cfu/mL was serially diluted 10-fold to 10^4^–10^8^ cfu/mL. Nucleic acids were extracted and analyzed as described above. All tests were performed in triplicate using the quantitative PCR protocol described previously on a LightCycler^®^ 480 platform. By plotting the logarithm of cell density (Log (cfu/mL)) as the x-axis and the Cq value as the y-axis, the standard curve and the linear regression equation were obtained. The Cq value of the sample was used to calculate the logarithm of cell density (X) using the equation. The amounts of *L. rhamnosus* X253 in the products were calculated using the formula below:

(1) $$M=10^{X}\times \left(A+B\right)/A\times C$$

where M is the amount of *L. rhamnosus* X253 in the products (cfu/g), X is the logarithm of cell density, A is the weighted mass of the test sample (g), B is the volume of the dilution solution (mL), and C is the dilution factor of the sample.

## 3. Results

### 3.1. SNP Analysis and Specificity

**Figure 1:**
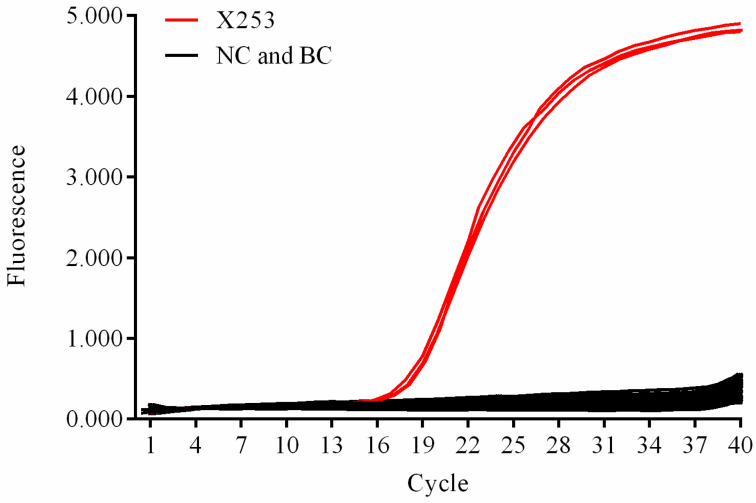
Specificity of the *Lacticaseibacillus rhamnosus* X253 strain-specific assay. NC: the strains other than X253, used as negative controls, BC: sterile water was used as a blank control.

Compared with 29 other *L. rhamnosus* strains, the genome of *L. rhamnosus* X253 was found to have a total of 12 strain-specific SNP loci (Table S1). Using quantitative PCR to amplify the *holA* gene SNP locus, *L. rhamnosus* X253 could be efficiently distinguished from the other 20 lactic acid bacteria. Cq values of 18.36, 18.61, and 18.65 were observed for *L. rhamnosus* X253, whereas no significant amplification was seen for all other strains or the blank control (Figure 1). The *holA* gene encodes the delta subunit of DNA polymerase III. According to Wang et al. [27], Cq values less than 35 are considered positive.

### 3.2. Stability during Passage

Using the above procedure, *L. rhamnosus* X253 was serially passaged and cultures of generations 5 to 19 were analyzed for Cq values. As shown in Table 4, the relative standard deviation was 0.41%, indicating that the selected SNP locus on the *holA* gene was relatively stable during the process of subculture and could be used to detect the strain of different generations with high accuracy.

### 3.3. Sensitivity and Efficiency

**Figure 2:**
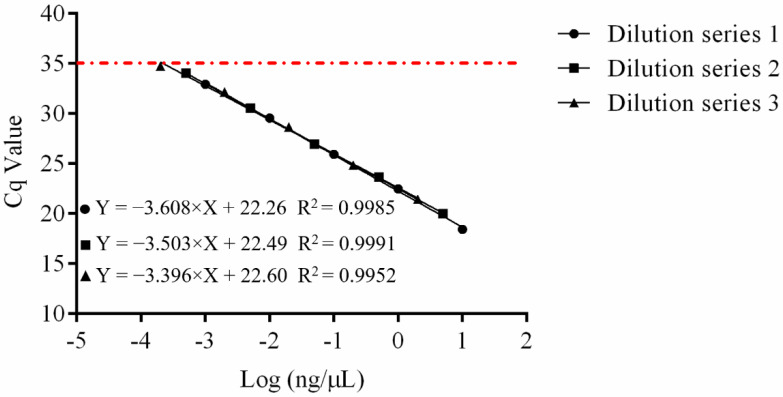
Sensitivity of the *Lacticaseibacillus rhamnosus* X253 strain-specific assay of DNA concentration. Three DNA solutions of 10, 5, and 2 ng/µL were subjected to a 10-fold dilution. The mean Cq values and logarithmic DNA concentration were used to create standard curves. The red line at the Cq value of 35 represented the limit of quantification. The slope values were −3.608, −3.503, and −3.396, and the reaction efficiency values were 89.3, 93.0, and 97.0%, respectively.

To determine the sensitivity of the assay, quantitative PCR amplification was done with serial dilutions of three initial nucleic acid solutions, 10, 5, and 2 ng/µL, respectively. (Figure 2 and Table S2). The slope values were −3.608, −3.503, and −3.396. Reaction efficiency values were 89.3, 93.0, and 97.0% and *R* squared values were 0.9985, 0.9991, and 0.9952. The sensitivity of the test was found to be 0.2 pg of genomic DNA, showing that the test was extremely sensitive.

**Figure 3:**
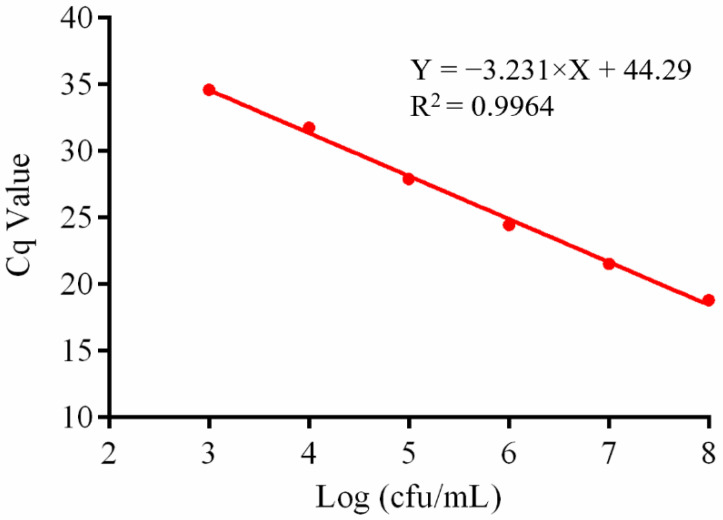
Sensitivity of the *Lacticaseibacillus rhamnosus* X253 strain-specific assay of cell density. The culture of *L. rhamnosus* X253 was serially diluted, and DNA was isolated for testing. The mean Cq values and logarithmic cell density were used to create standard curve. The slope value was −3.231, and reaction efficiency value was 103.9%.

To correlate quantitative data with colony-forming units, DNA was extracted from 1 mL of *L. rhamnosus* X253 culture with cell densities ranging from 10^8^ to 10^2^ cfu/mL and examined using quantitative PCR. The results revealed that all tests were positive for samples, with cell densities higher than or equivalent to 10^3^ cfu/mL (Figure 3 and Table S3). A standard curve was created with the logarithm of cell density against the Cq value. The slope value was −3.231, the reaction efficiency value was 103.9%, and the R squared value was 0.9964, indicating a good linear regression. As a result, the method was found to be sensitive to a cell density of 10^3^ cfu/mL, which satisfied the requirement for most probiotic products, where the cell density of probiotic bacteria was commonly higher than 10^6^ cfu/mL [28]. Due to the cut-off value, all observed reaction efficiencies were more than 89%, which was within the optimal range (80–120%) [29]. The high R squared value indicates good linearity of the assay [29].

### 3.4. Repeatability and Reproducibility

**Figure 4:**
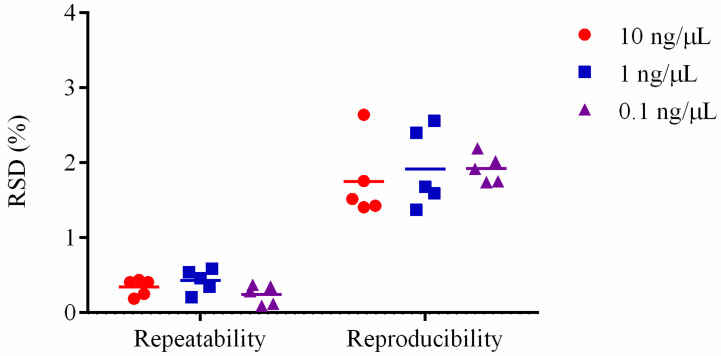
Repeatability and reproducibility of the *Lacticaseibacillus rhamnosus* X253 strain-specific assay. Five samples were utilized, each with a different DNA concentration (0.1, 1, and 10 ng/µL). The experiment was repeated over a short period of time to assess repeatability and was performed on two quantitative PCR platforms (LightCycler^®^ 480 and ABI7500) to assess reproducibility. Relative standard deviation (RSD) is used to express repeatability and reproducibility.

Five X253 samples were utilized to assess repeatability and reproducibility, with each sample being evaluated at three different DNA concentrations (0.1, 1, and 10 ng/µL). RSD of repeatability and reproducibility is especially important in quantitative PCR tests, where it should be less than or equal to 25% [29]. RSD of repeatability for *holA*-based quantification was found to vary from 0.19 to 0.44% at 10 ng/µL, 0.21 to 0.59% at 1 ng/µL, and 0.09 to 0.37% at 0.1 ng/µL (Figure 4 and Table S4). RSD of reproducibility ranged from 1.41 to 2.64% at 10 ng/µL, 1.37 to 2.56% at 1 ng/µL, and 1.74 to 2.19% at 0.1 ng/µL (Figure 4 and Table S5). The results showed that the assay worked well in terms of repeatability and reproducibility and was adaptable to different quantitative PCR platforms.

### 3.5. Assay of the Spiked Sample

**Figure 5:**
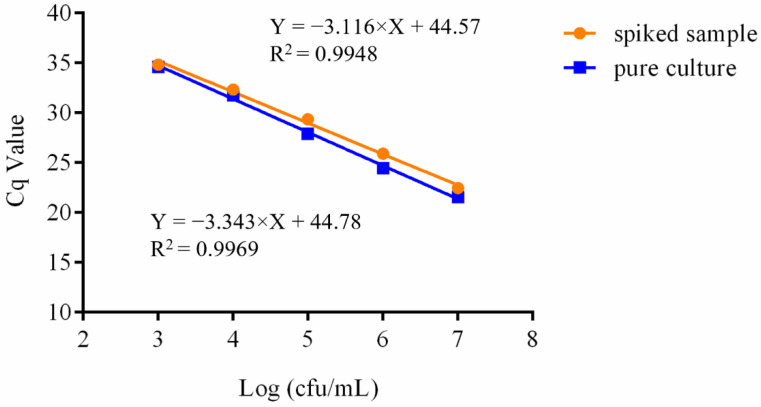
Development of standard curves in pure culture and spiked samples for the *Lacticaseibacillus rhamnosus* X253 strain-specific assay. Each point represents the mean Cq value of the quantitative PCR. The slopes were −3.343 and −3.116 for pure culture and spiked sample, respectively.

To verify the feasibility of this approach to test actual dairy samples, X253 culture was serially diluted in a substrate of multi-strain fermented milk and used as spiked samples for X253 quantification. As shown in Figure 5, high linearity and a detection limit of 10^3^ cfu/mL were seen. The results showed that adding other strains and substrates had no significant effect on the detection limit of this assay (Table S6), so it could be used to detect X253 in actual dairy products.

### 3.6. Assay of the Actual Sample

**Figure 6:**
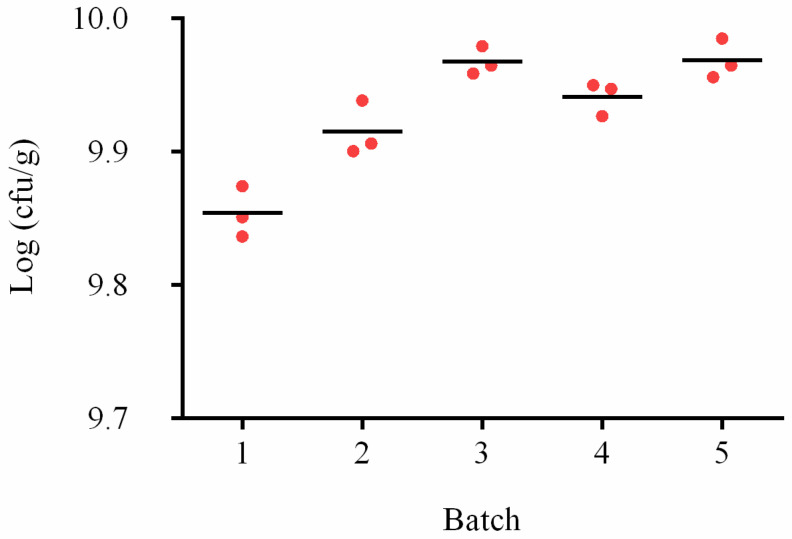
*Lacticaseibacillus rhamnosus* X253 strain-specific assay in actual powder samples. Five batches of multi-strain bacterial powder samples, including oligosaccharides and excipients, were employed in the actual product assay. Each batch of samples was tested three times (red dot). The powder was made up of *L. rhamnosus* X253, *Bifidobacterium animalis subsp. lactis* Bb12, *L. paracasei* N115, and *L. acidophilus* NCFM.

Five batches of actual product samples with X253 were assayed to determine the number of X253 cells (Table S7). Using the standard curve (Table S8), the number of *L. rhamnosus* X253 cells was calculated to be within a range of 9.85±0.02 to 9.97±0.01 Log (cfu/g) for five batches (Figure 6). These results showed that the proposed approach was suitable for accurately detecting an individual strain in the presence of substrates and other strains with strong interference resistance, while plate counts were unable to measure a specific strain in a multi-strain sample.

## 4. Discussion

*L. rhamnosus* strains GG and HN001 are commonly regarded as harmless, and their advantageous properties have been well investigated [30,31]. They are commonly applied to dairy products [32], animal feeds [33], and biopharmaceuticals [34]. In recent work, we found that *L. rhamnosus* X253 demonstrated a much higher tolerance to hydrogen peroxide than *L. rhamnosus* GG. In addition, the X253 strain could use sucrose and lactose, which were unavailable to the *L. rhamnosus* GG strain. To better evaluate the efficacy of probiotic strains, strain-level testing of probiotic bacteria is a key issue to be addressed.

Some PCR-based approaches were used to identify *L. rhamnosus* at the strain level. Ahlroos et al. used RAPD to identify a 700-bp region targeting a transposase gene of *L. rhamnosus* GG and detected *L. rhamnosus* GG in human fecal samples [14]. Brandt et al. successfully identified *L. rhamnosus* GG using PCR primers based on phage Lc-Nu regions [35]. Zhang et al. analyzed the genomes of *L. rhamnosus* LV108 and *L. rhamnosus* hsryfm 1301 to identify particular segments and then utilized PCR with various primers to identify the target strains at the strain level [36]. These tests need further processing of PCR products, such as gel visualization and sequencing. In comparison, quantitative PCR is advantageous since it enables quantitative monitoring of reactions. The challenge is to come up with efficient quantitative PCR primers and probes for measuring at the strain level.

With the advent of high-throughput sequencing, an increasing number of *L. rhamnosus* genomes have been sequenced, and many of them are now available via NCBI and other public databases. In the realm of probiotics, SNP analysis mostly involves the typing of *Lacticaseibacillus* and *Bifidobacterium*. However, there is a lack of criteria for the identification of probiotic strains [16]. In this study, by comparing the genome of *L. rhamnosus* X253 to those of 29 other *L. rhamnosus* strains (Table 1), a total of 12 specific SNP loci were found, from which a particular locus on the *holA* gene (Table 2) was proven to be efficient in distinguishing *L. rhamnosus* X253 from others (Table 3) and remained stable over serial passages (Table 4). The *holA* gene encodes the delta subunit of DNA polymerase III. DNA polymerase III participates in several DNA metabolic activities during replication and repair, and the delta subunit is likely engaged in beta clamp recycling during DNA replication [37]. However, when more genomic data are available for *L. rhamnosus* and the closely related species like *L. chiayiensis* and *L. zeae*, it will be more difficult to identify a particular strain using a single SNP analysis. Multiple SNP loci-based analysis might be essential for the accurate identification of strain X253.

In this study, we used a pipeline with a Cq value of 35 for the majority of qPCR analyses, where Cq values greater than 35 indicated no amplification [38]. According to the regression equation, the limit of quantification for the nucleic acid concentration was calculated to be 0.00029, 0.00027, and 0.00022 ng/µL, for Dilution series 1, 2, and 3 respectively, which are essentially the same (Figure 2). The sensitivity of bacterial cell density was 10^3^ cfu/mL whether different probiotic bacteria and substrates were added, demonstrating that the approach was suited for detecting strain X253 in complex strains (Figure 3 and Figure 5). However, this result is based on DNA extracted from different bacterial cell densities and cannot distinguish between dead and living bacterial cells. To identify strain-specific live bacteria, the approach needs to be supplemented with reactive dyes such as propidium monoazide in future studies [39].

Additionally, the repeatability of five samples at three distinct DNA concentrations (10, 1, and 0.1 ng/µL) were assessed with less than 1% variance. The assay could be performed on different platforms (LightCycler^®^ 480 and ABI7500) with less than 3% variance (Figure 4). In general, the majority of probiotics added to products are complex organisms, and it is essential to prevent interference from other strains and substances. The strain-specific approach was successfully applied to artificially spiked milk samples (Figure 5) and multi-strain powder samples (Figure 6), which might be used for diverse product matrices, such as nutritional supplements and food. Future studies into the application of shelf-life stability of live bacteria will boost the approach.

## 5. Conclusions

The assay developed for strain-specific detection of the *L. rhamnosus* X253 strain has a high degree of specificity, stability, sensitivity, optimal reaction efficiency, and low variance. Additionally, the approach can be performed on different quantitative PCR platforms and used to assess the colony-forming units in multi-strain dairy products. This approach, combining SNP analysis with quantitative PCR, provides a feasible method for strain-specific identification and enumeration in dairy products for managing quality control and quality assurance.

## Figures and Tables

**Table 1:** Strains used for genomic comparison.

Species	Strain	Assembly No.	Scaffold Number	Genome Size (Mb)
*L. rhamnosus*	X253 (ref)	GCA_018228745.1	1	2.99
	1.032	GCA_006151905.1	1	2.94
	4B15	GCA_002158925.1	1	3.05
	ATCC 11443	GCA_003433395.1	1	2.99
	ATCC 8530	GCA_000233755.1	1	2.96
	B6	GCA_016599675.2	1	2.92
	BFE5264	GCA_001988935.1	2	3.11
	BIO5326	GCA_009720565.1	1	2.99
	BIO6870	GCA_008831425.1	1	3.01
	BPL5	GCA_900070175.1	1	3.02
	DSM 14870	GCA_002287945.1	1	3.01
	GG (ATCC 53103)	GCA_000026505.1	1	3.01
	HN001	GCA_000173255.2	96	2.91
	hsryfm 1301	GCA_008727835.1	2	3.07
	JL-1	GCA_015238575.1	1	3.01
	KF7	GCA_016653515.1	4	3.25
	Lc 705	GCA_000026525.1	2	3.03
	LOCK900	GCA_000418475.1	1	2.88
	LOCK908	GCA_000418495.1	1	2.99
	LR5	GCA_002286235.1	1	2.97
	LRB	GCA_001721925.1	1	3.01
	LR-B1	GCA_004010975.1	1	2.92
	LV108	GCA_013167115.1	1	3.01
	MGYG-HGUT-01293	GCA_902381635.1	1	2.99
	NCTC13710	GCA_900636875.1	1	2.99
	NCTC13764	GCA_900636965.1	94	2.98
	Pen	GCA_002076955.1	1	2.88
	R0011	GCA_000235785.2	10	2.90
	SCT-10-10-60	GCA_002960215.1	1	2.99
	TK-F8B	GCA_015377485.1	3	3.06

**Table 2:** Sequences of primers and probe.

Gene	Primers and Probe	Length (bp)
*holA*	GGTTGGTCGTTTGCCTTATCA	61
	TTCAGTATCCACCAGCCCACTA	
	FAM-ACTGGCCCATGCTT-BHQ1	

Note: The SNP locus on the probe is labelled in red.

**Table 3:** Strains used for specificity tests.

Species	Strain	Source
*Lacticaseibacillus rhamnosus*	X253	Isolated
	GG	Danisco
	HN001	Danisco
	ATCC 7469	CICC ^1^
	ATCC 8530	CICC
	ATCC 11443	CICC
*Lacticaseibacillus casei*	Lc-11	Danisco
*Lacticaseibacillus paracasei*	Lpc-37	Danisco
*Bifidobacterium animalis subsp. lactis*	Bb-12	Danisco
	Bi-07	Danisco
	HN019	Danisco
	DSMZ 10140	Danisco
*Lactobacillus acidophilus*	NCFM	Danisco
	La-14	Danisco
	AS1.2686	Danisco
	Lp-115	Danisco
*Lactococcus lactis subsp. lactis*	CICC 6246	CICC
*Lactococcus lactis subsp. cremoris*	CICC 20407	CICC
*Lactococcus lactis subsp. hordniae*	CICC 21034	CICC
*Ligilactobacillus salivarius*	Ls-33	Danisco
*Lactobacillus delbrueckii subsp. bulgaricus*	CICC 6097	CICC

^1^: China Center of Industrial Culture Collection.

**Table 4:** Stability of specific amplification during serial passages.

Number of Generations	Cq			Mean Cq	RSD (%)
5	18.63	19.40	18.70	18.91	0.41
9	18.77	18.71	19.51	19.00	
15	19.27	18.69	18.63	18.86	
19	18.81	18.69	18.95	18.82	

## Data Availability

The full genome data of *L. rhamnosus* X253 can be found in NCBI GenBank under the accession number CP073711.

## Supplementary Materials

The following supporting information can be downloaded at: https://www.mdpi.com/article/10.3390/foods11152282/s1, Table S1: Strains used for genomic comparison and specific loci information; Table S2: Results for analytical sensitivity testing of DNA concentration; Table S3: Results for analytical sensitivity testing of cell density; Table S4: Results for repeatability testing; Table S5: Results for reproducibility testing; Table S6: Results for pure culture and spiked sample testing; Table S7: Results for actual sample testing; Table S8: Results for the standard curve of cell density in the actual sample testing.
